# Cellular phosphoinositides and the maturation of bluetongue virus, a non-enveloped capsid virus

**DOI:** 10.1186/1743-422X-10-73

**Published:** 2013-03-05

**Authors:** Bishnupriya Bhattacharya, Polly Roy

**Affiliations:** 1 Faculty of Infectious and Tropical Diseases, London School of Hygiene and Tropical Medicine, Keppel Street, WC1E 7HT, London, UK

**Keywords:** BTV, Lipids, PI(4,5)P_2_, Assembly, Maturation, NS3, Membrane

## Abstract

**Background:**

Bluetongue virus (BTV), a member of *Orbivirus* genus in the *Reoviridae* family is a double capsid virus enclosing a genome of 10 double-stranded RNA segments. A non-structural protein of BTV, NS3, which is associated with cellular membranes and interacts with outer capsid proteins, has been shown to be involved in virus morphogenesis in infected cells. In addition, studies have also shown that during the later stages of virus infection NS3 behaves similarly to HIV protein Gag, an enveloped viral protein. Since Gag protein is known to interact with membrane lipid phosphatidylinositol (4,5) bisphosphate [PI(4,5)P_2_] and one of the known binding partners of NS3, cellular protein p11 also interacts with annexin a PI(4,5)P_2_ interacting protein, this study was designed to understand the role of this negatively charged membrane lipid in BTV assembly and maturation.

**Methods:**

Over expression of cellular enzymes that either depleted cells of PI(4,5)P_2_ or altered the distribution of PI(4,5)P_2_, were used to analyze the effect of the lipid on BTV maturation at different times post-infection. The production of mature virus particles was monitored by plaque assay. Microscopic techniques such as confocal microscopy and electron microscopy (EM) were also undertaken to study localization of virus proteins and virus particles in cells, respectively.

**Results:**

Initially, confocal microscopic analysis demonstrated that PI(4,5)P_2_ not only co-localized with NS3, but it also co-localized with VP5, one of the outer capsid proteins of BTV. Subsequently, experiments involving depletion of cellular PI(4,5)P_2_ or its relocation demonstrated an inhibitory effect on normal BTV maturation and it also led to a redistribution of BTV proteins within the cell. The data was supported further by EM visualization showing that modulation of PI(4,5)P_2_ in cells indeed resulted in less particle production.

**Conclusion:**

This study to our knowledge, is the first report demonstrating involvement of PI(4,5)P_2_ in a non-enveloped virus assembly and release. As BTV does not have lipid envelope, this finding is unique for this group of viruses and it suggests that the maturation of capsid and enveloped viruses may be more closely related than previously thought.

## Background

Bluetongue virus (BTV), a vector-borne animal pathogen has recently emerged in Europe causing high mortality in sheep. BTV is prototype of Orbivirus genus of the *Reoviridae* family. Like other family members, BTV is a non-enveloped icosahedral particle and is composed of seven structural proteins (VP1-VP7) organized in two concentric capsids [1]. BTV enters the cells via receptor-mediated endocytosis and the two outer capsid proteins, VP2 and VP5 are involved in cell attachment and membrane penetration [2-6]. Although the membrane penetration protein VP5 is non-glycosylated, structurally it resembles the glycosylated fusion proteins of enveloped viruses, such as HIV, herpesviruses, vesicular stomatitis virus and influenza virus [7]. The inner capsid or “core,” is comprised of the remaining five proteins, two major (VP7 and VP3), three minor enzymatic (VP1, VP4, VP6) and a genome of ten double-stranded RNA (dsRNA) segments. In addition, BTV also synthesizes four non-structural proteins (NS1, NS2, NS3/NS3A, NS4) in infected cells, of which the small NS3 protein is glycosylated. Upon infection, the core particles become active, synthesizing ten capped single-stranded RNA transcripts (ssRNAs) which extrude through the capsid pores into the cytoplasm. The newly synthesized core components are recruited by NS2, triggering the formation of virus-specific inclusion bodies (VIBs), the site of the core assembly [8,9]. The addition of newly synthesized VP2 and VP5 onto the cores does not occur within VIBs [8,10]. Instead these two proteins appear to be associated with NS3, the only protein of BTV that is glycosylated. NS3 has been localized to intracellular organelles (Golgi complex and Endoplasmic reticulum), cellular membranes and is associated with virus release [11-14]. It also interacts with Tsg101 [13,14], a component of multivesicular bodies (MVBs) and with cellular protein p11 that forms a complex with annexin 2 [15,16], a member of the cellular exocytotic pathway. Although it has been demonstrated that NS3 localizes to cellular membranes, the cellular components responsible for targeting NS3 to the cellular membrane have not yet been defined. Annexin-2, a binding partner of p11 has been demonstrated to interact with Phosphatidylinositol (4,5) bisphosphate [PI(4,5)P_2_], a negatively charged lipid molecule in cellular membranes [17-22]. It is known that PI(4,5)P_2_ also interacts with members of the SNARE (soluble N-ethylmaleimide sensitive fusion protein receptors) superfamily [23]. Interestingly, while NS3 binds p11, the outer capsid protein VP5 possesses a SNARE domain [24] indicating that BTV NS3 and VP5 may use these cellular components during virus morphogenesis.

The membrane lipid PI(4,5)P_2_ belongs to a family of lipid molecules that is collectively known as phosphoinositides [25]. These lipid molecules are generally inter-converted by specific cellular lipid phosphatases and kinases. While the level of PI(4,5)P_2_ in cells is maintained by phosphatases such as polyphosphoinositide 5-phosphatase (5ptaseIV), a cellular kinase, namely phosphatidylinositol-4-phosphate 5-kinase generates the majority of PI(4,5)P_2_ in cells. More importantly, this cellular kinase itself is regulated by a number of factors including the small G protein ADP-ribosylation factor 6 (Arf6) [26]. It is known that the expression of a constitutively active form of Arf6, defective for GTP hydrolysis (Arf6/Q67L), alters the localization of cellular PI(4,5)P_2_ by inducing the formation of PI(4,5)P_2_-enriched endosomal structures [27,28]. Since annexin-2 and SNARE domains interact with PI(4,5)P_2,_ and BTV has been shown to use similar egress machinery to HIV [13,14], this current study was undertaken to investigate whether the membrane lipid PI(4,5)P_2_ plays any role in BTV maturation and assembly as it does in HIV.

For this purpose we used a combination of molecular, biochemical and microscopic techniques to investigate the effect of PI(4,5)P_2_ on BTV maturation. We found that when the level of PI(4,5)P_2_ was reduced by over expression of 5ptaseIV, the virus titres were also decreased significantly. Furthermore, BTV growth was also affected when PI(4,5)P_2_ distribution was altered to form cellular vesicles using a plasmid that expresses an Arf6 mutant (Arf6/Q67L). The results obtained strongly suggest that PI(4,5)P_2_ plays a key role in localizing BTV to cellular membranes and promotes efficient virus production. This observation is the first demonstration of the importance of membrane lipids in the morphogenesis of a non-enveloped virus.

## Results

### BTV proteins associate with PI(4,5)P_2_ in infected cells

**Figure 1:**
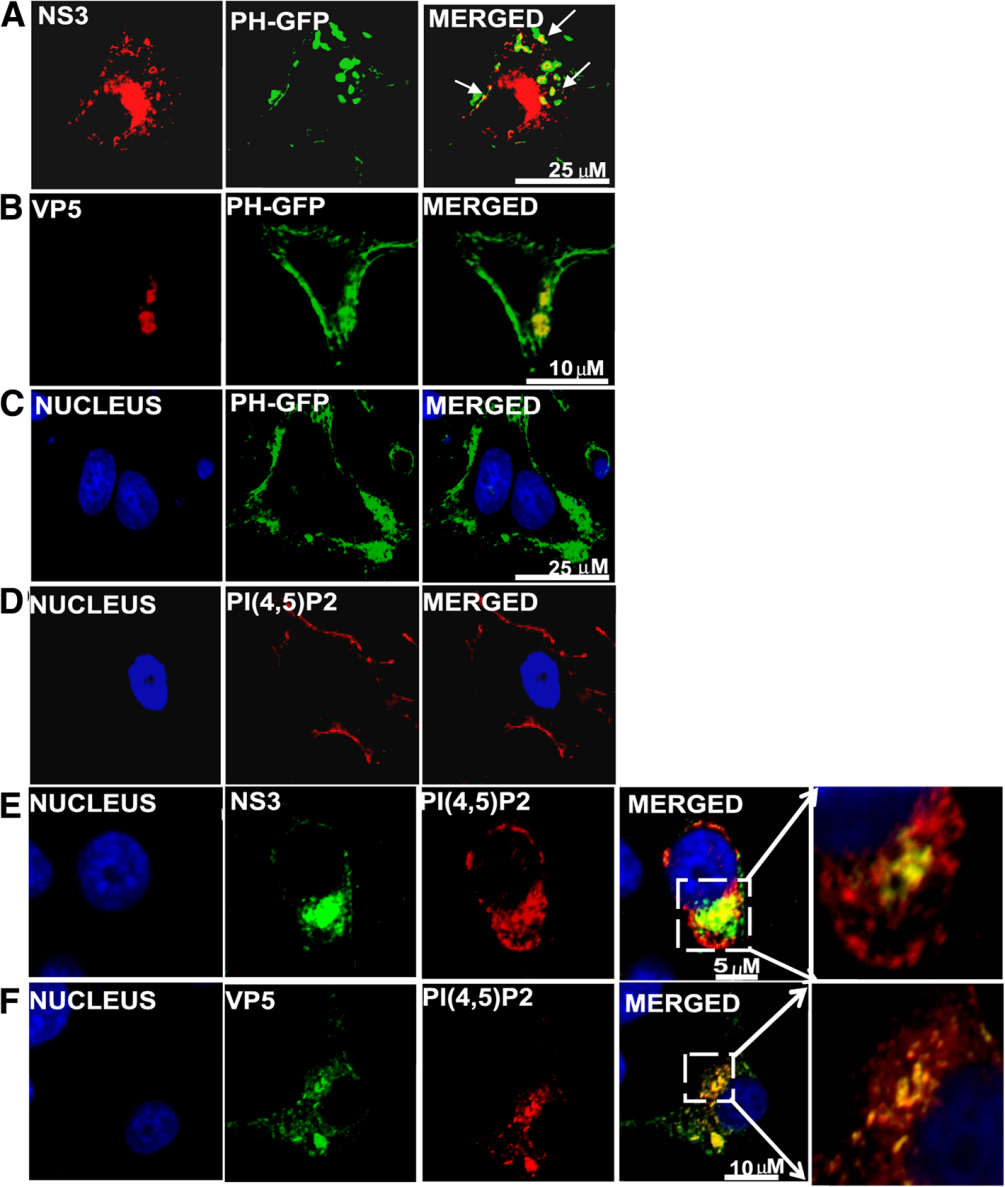
**Immunofluorescence analysis of NS3 and VP5 co-localization with PI(4,5)P**_**2 **_**in BTV infected cells.** HeLa cells either transfected with plasmid expressing PH-GFP (green)(**B-C**) or not transfected (**E-F**), were infected with BTV-1 and analyzed for localization with NS3 (**A,E**) and VP5 (**B,F**) 12hrs post-infection. Control consists of uninfected cells either expressing PH-GFP (**C**) or cells not expressing PH-GFP, but immulolabelled for PI(4,5)P_2_(**D**) (red). Area of co-localization shown by yellow has been magnified in the extreme right panel (**E-F**). In all the panels the nucleus (blue) was stained by Hoechst. The viral proteins (NS3 and VP5) were stained were either stained with TRITC (red) (**A-B**) or with FITC (green) (**E-F**). Small white arrows indicate the areas of co-localization shown by yellow. The scale has been included in the merged panel of each row.

We have shown previously that while BTV outer capsid protein VP5 possesses a SNARE domain [24], the non-structural glycoprotein NS3 has functional similarities with the Gag protein of HIV [13,14]. Since it is known that both HIV Gag and SNARE proteins interact with PI(4,5)P_2,_[23] we hypothesized that PI(4,5)P_2_ might be involved in BTV life cycle. To investigate this, we first examined if VP5 and NS3 in BTV-infected cells are co-localized with PI(4,5)P_2_. BTV infected HeLa cells expressing the pleckstrin homology (PH) domain of phospholipase Cδ1 tagged with GFP (PH-GFP) [29] demonstrated co-localization of PH-GFP with NS3 (Figure 1A) (71.33% ± 1.9) and VP5 (Figure 1B) both on the plasma membrane and in the cytoplasmic vesicular-like structures. In contrast, when PH-GFP was expressed alone in uninfected HeLa cells, it was localized primarily on the plasma membrane (Figure 1C). Further, when a specific monoclonal antibody was used to assess localization of these two BTV proteins with membrane PI(4,5)P_2_ in BTV infected cells, distinct co-localization of NS3 (Figure 1E) and VP5 (Figure 1F) with PI(4,5)P_2_ were also clearly visible. As before, uninfected control cells showed PI(4,5)P_2_ expression only on the plasma membrane (Figure 1D). These results indicate that PI(4,5)P_2_ co-localizes with the viral outer capsid protein VP5 as well as with the glycosylated NS3 in BTV infected cells.

### BTV particle production is affected when cellular PI(4,5)P_2_ level was perturbed

**Figure 2:**
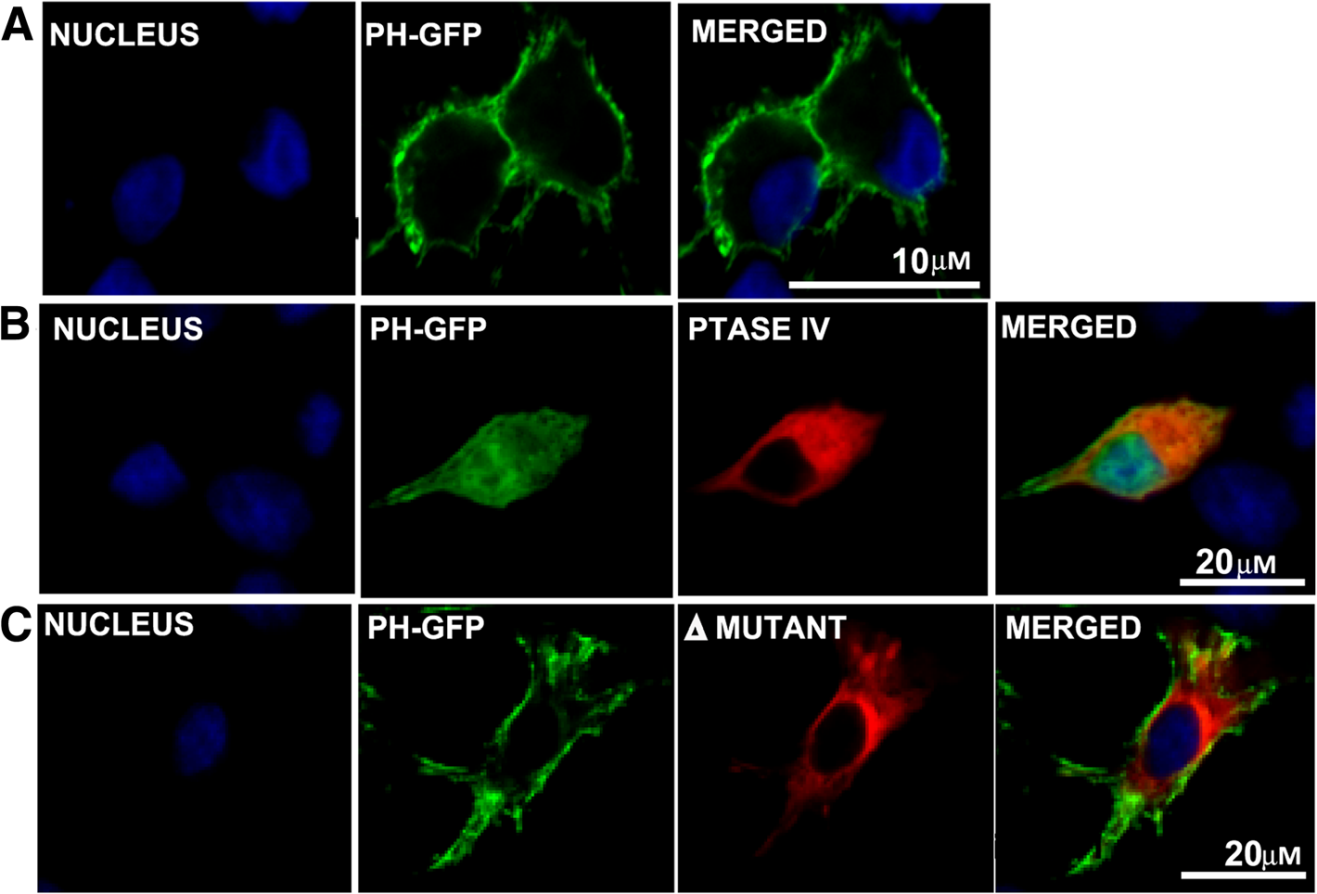
**Effect of 5ptaseIV expression on PI(4,5)P**_**2 **_**expression.** HeLa cells were transfected with PH-GFP (**A**) or co-transfected with PH-GFP and myc-tagged 5ptaseIV (red) (**B**) or the myc-tagged Δ1 mutant (red) (**C**). The scale has been included in the merged panel of each row.

**Figure 3:**
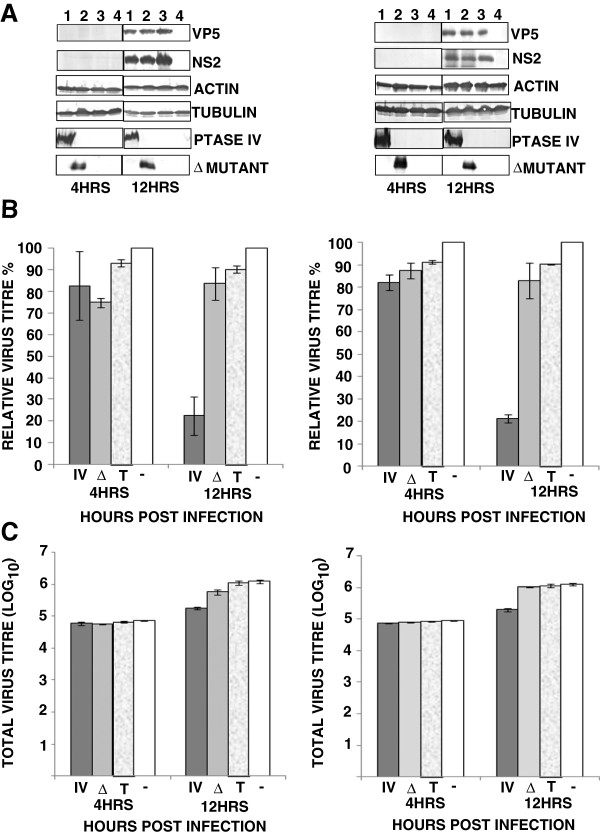
**Depletion of PI(4,5)P**_**2 **_**and it’s effect on BTV production.** (**A**) Western Blot analysis of HeLa (right) and BSR (left) cells transfected with myc-tagged 5ptaseIV (lane 1) or Δ1 mutant (lane 2) and infected with BTV-1 for the expression of viral (VP5 and NS2) and cellular (actin and tubulin) proteins 4 and 12 hrs post-infection. Controls consist of HeLa cells that have not been transfected but infected with BTV-1 (3) and cells that have neither been transfected nor infected (4). The proteins were detected by specific antibodies. The western blots were repeated 2 times on three independent experiments. (**B** and **C**) Analysis of relative (**B**) and total (**C**) virus titres in infected HeLa (right) and BSR (left) cells in the presence of 5ptaseIV (IV), Δ mutant (Δ), transfection reagent only (T) or absence of any plasmid (−). Cells infected with BTV-1, 12 hrs post-transfection with either 5ptaseIV or Δ mutant were harvested at 4 and 12 hrs post-infection. For the relative virus tires, the total titre for each time post-infection was normalized to 100% for untreated cells. Bars indicate the standard error of three replicates of the experiments.

Since Phosphatases such as 5ptaseIV have been shown to reduce the cellular level of PI(4,5)P_2_[30], we examined if presence of 5ptaseIV alters the distribution of PI(4,5)P_2_ in cells. On transfecting the cells with 5ptaseIV expressing plasmids [31], the distribution of PH-GFP was visibly altered from the plasma membrane (Figure 2A) to a diffuse expression throughout the cytoplasm (Figure 2B). The experiment was repeated three times and in each experiment, a majority of the cells (~85% in three different planes) expressing 5ptaseIV showed a diffused expression of PH-GFP suggesting that 5ptaseIV does have an effect on the altered expression of PI(4,5)P_2_. In comparison, control cells co-transfected with a mutated version of 5ptaseIV(Δ1) lacking the 5-phosphatase signature domain (Δ1 mutant ) showed no effect on the localization of PH-GFP and the pattern was similar to that of panel ‘A’ (compare Figure 2A with Figure 2C). To further examine the effect of PI(4,5)P_2_ depletion on virus growth and protein production, two different type of mammalian cells (HeLa and BSR) were transfected in parallel with the 5ptaseIV or Δ1 mutant expression plasmids. HeLa and BSR cells expressing myc-tagged 5ptaseIV (>60%) or Δ1 mutant (>60%) were infected with BTV-1 for 4 or 12 hours (hrs) and VP5, a major outer capsid protein synthesis was examined in both cells (Figure 3A) by western analysis. The results were confirmed by repeating the experiments three times and Western Blot was conducted twice per experiment. In addition, NS2, a major non-structural BTV protein, which is involved in core particle assembly [8,10,32], was used to monitor overall viral replication. Viral protein expressions were observed at 12 hrs, but not at 4 hrs post-infection in BTV-1 infected cells expressing either 5ptaseIV (Figure 3A right & left panels, lane 1) or the Δ1 mutant (Figure 3A right & left panels, lane 2). Infected control cells, not expressing any plasmid, also showed expression of viral proteins at 12 hrs but not at 4 hours post infection (Figure 3A right & left panels, lane 3). Moreover, expression of cellular proteins (tubulin and actin) was monitored in each experiment: in depleted and infected cells (Figure 3A right & left panels, lane 1); not depleted and infected cells (Figure 3A right & left panels, lane 2); control normal cells that have been infected (Figure 3A right & left panels, lane 3) and uninfected cells (Figure 3A right & left panels, lane 4). These data confirmed that cellular depletion of PI(4,5)P_2_ did not significantly alter the production of cellular proteins. In all experiments the expression of myc-tagged 5ptaseIV (Figure 3A right & left panels, lane 1) and Δ1 mutant (Figure 3A right & left panels, lane 2) expression plasmids were also monitored. Further, infected cells treated with transfection reagent also showed similar expression profiles of the proteins as the control infected cells without any transfection (data not shown).

Subsequently, to investigate whether the depletion of PI(4,5)P_2_ hinders virus assembly, the total virus titres of the post-transfected cells infected with BTV were determined at 4 and 12 hrs. When viral titres at each time point were plotted either as the relative percentages of the titres in infected cells that were not transfected but infected (Figure 3B), or as total titres (Figure 3C), the virus titres in the cells over expressing 5ptaseIV were significantly reduced at 12 hrs post-infection in both HeLa (p = 0.008 and 0.003 in Figure 3B and 3C right, respectively) and BSR (p = 0.003 and 0.001 in Figure 3B and 3C left, respectively) cells. In contrast the reduction in virus titres of infected cells expressing Δ1 mutant was not significant either when the titres were plotted as a relative percentage (Figure 3B) of infected but not transfected cells (p = 0.07 in both HeLa and BSR cells) or as total titres (p = 0.1 and 0.2 in HeLa and BSR cells, respectively) (Figure 3C). Thus, depletion of PI(4,5)P_2_ inhibits virus titres but does not interfere with virus protein production in infected cells at 12 hrs. In order to negate the deleterious effect of the transfection reagent on virus replication, cells treated with only transfection reagent were also infected with BTV. There was no noticeable difference in virus titres between the cells that were transfected with plasmids or transfection reagent (data not shown) prior to infection.

**Figure 4:**
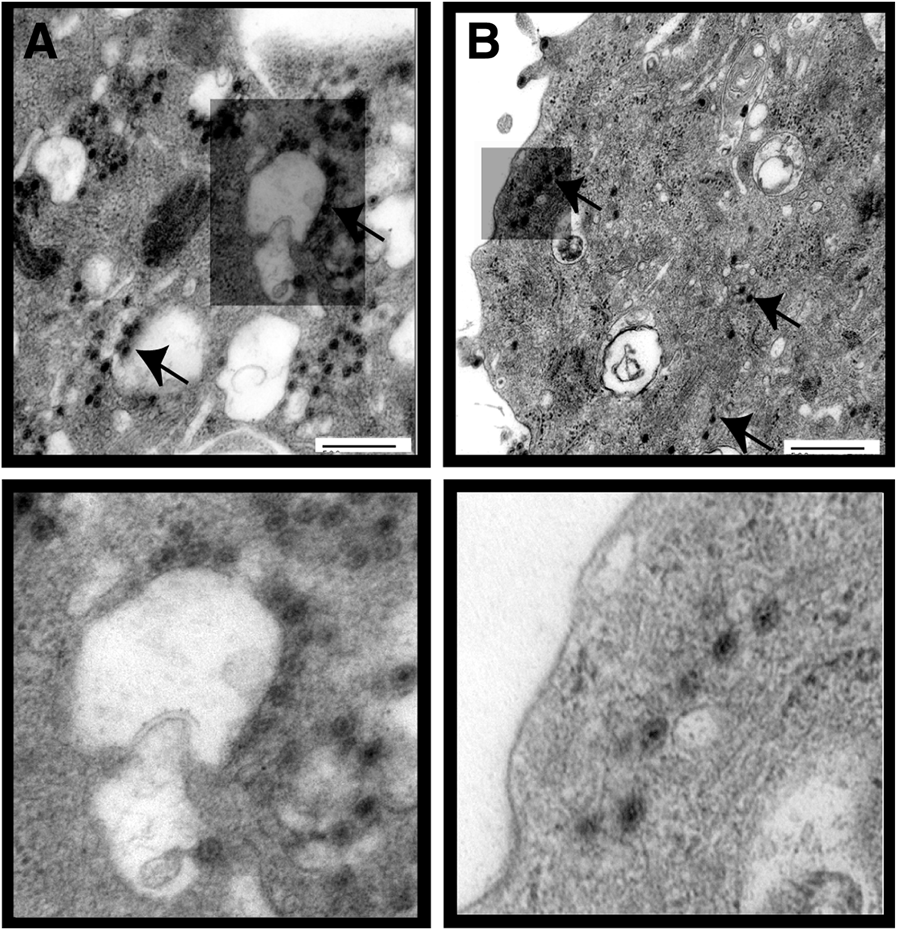
**EM analysis of cells depleted of PI(4,5)P**_**2**_**.** HeLa (**B**) cells depleted of PI(4,5)P_2_ by transient expression of 5ptaseIV were infected with BTV-1 and analysed for virus particle production. Controls (**A**) consist of HeLa cells that have not been depleted of PI(4,5)P_2_ but infected with BTV-1. The presence of virus particles in cell section (upper panel) is indicated by arrows. Lower panels are magnified section. The bars represent magnification of 500 nm.

Since the depletion of PI(4,5)P_2_ affected relative virus production, the distribution of viral particles in these cells was visualized by EM (Figure 4). Synthesis of virus particles in the PI(4,5)P_2_ depleted HeLa cells by ptase IV expression was decreased significantly (~86%, p = 0.002) in comparison to that of the control cells (compare Figure 4B with A). When the infected and transfected cells were scored in 3 different planes for the presence of virus particles around intra-cytoplasmic vesicles hardly any of the total counted particles bordered these structures. In contrast, control infected cells (Figure 4A) that were not depleted of PI(4,5)P_2_ exhibited the presence of virus particles attached to the outer surface of the vesicle-like structures in both type of cells. Thus, the EM data further support the hypothesis that PI(4,5)P_2_ expression in cells plays an important role in not only BTV particle production but also in the distribution of particles in infected cells.

### Changing the normal distribution of PI(4,5)P_2_ decreases virus particle production

**Figure 5:**
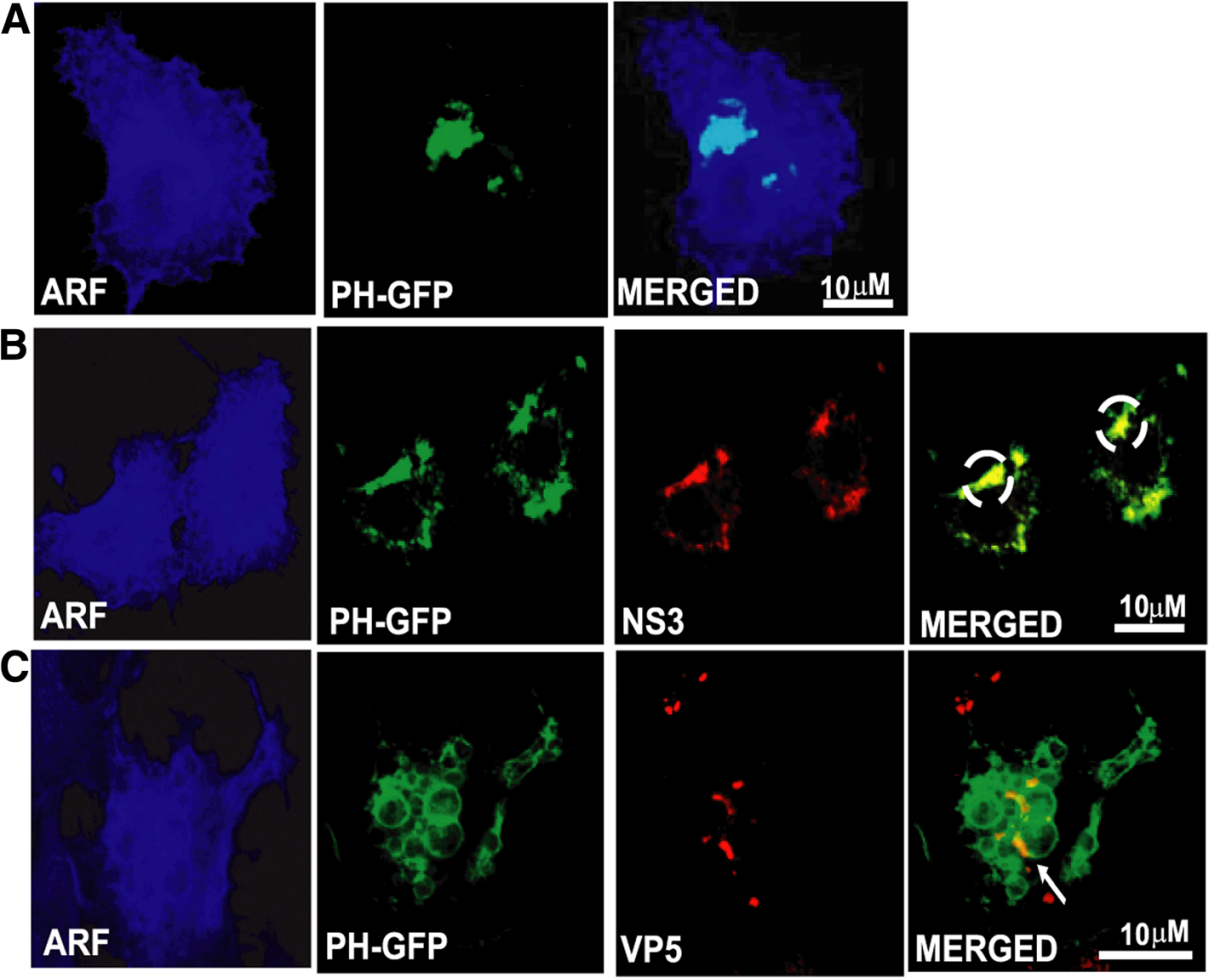
**Sequestration of virus proteins in Arf6/Q67L induced PI(4,5)P**_**2**_**-enriched vesicles.** Immunofluorescence microscopy of HeLa cells expressing HA-tagged Arf6/Q67L and PH-GFP followed by BTV-1 infection. The infected cells were analyzed 12hrs post-infection for localization of NS3 (**B**) and VP5 (**C**). Control consists of cells that have been co-transfected with PH-GFP and HA-tagged Arf6/Q67L, but not infected (**A**). While PH-GFP localization is shown in green, HA-tagged Arf6/Q67L was detected with Alexa Fluor (blue) and all the viral proteins with TRITC (red). White circles point to co-localized patches. The scale has been included in the merged panel of each row.

Since 5ptaseIV not only dephosphorylates PI(4,5)P_2_, but also dephosphorylates PI(3,4,5)P_3_[33]_,_ we designed experiments to nullify the effect of PI(3,4,5)P_3_ dephosphorylation on BTV replication. For this purpose constitutively active Arf6/Q67L that regulates the activity of phosphatidylinositol-4-phosphate 5-kinase [26,34] and induces the accumulation of PI(4,5)P_2_-enriched endosomal structures [27,28,31] was utilized. The effect of PI(4,5)P_2_ sequestration on BTV replication was first analyzed by co-transfecting HeLa cells with Arf6/Q67L and PH-GFP expression plasmids. Subsequently the cells were infected with BTV-1 and expression of NS3 and VP5 in these cells was monitored by confocal microscopy at 12 hrs post-infection (Figure 5). NS3 expression was not only restricted to the vesicle-like structures formed in the cells expressing Arf6/Q67L, but co-localization was also visualized between PH-GFP, a PI(4,5)P_2_ marker and NS3 (Figure 5B). In the case of VP5, the viral protein was visible in the vicinity of the vesicle-like structures formed due to PI(4,5)P_2_ sequestration (Figure 5C). Since control transfected (Arf6/Q67L and PH-GFP) cells that were not infected also demonstrated presence of PH-GFP only in intra-cytoplasmic vesicle-like structures (Figure 5A), these results suggested that Arf6/Q67L influenced PI(4,5)P_2_ accumulation in intracellular vesicles shifted NS3 localization to these structures.

**Figure 6:**
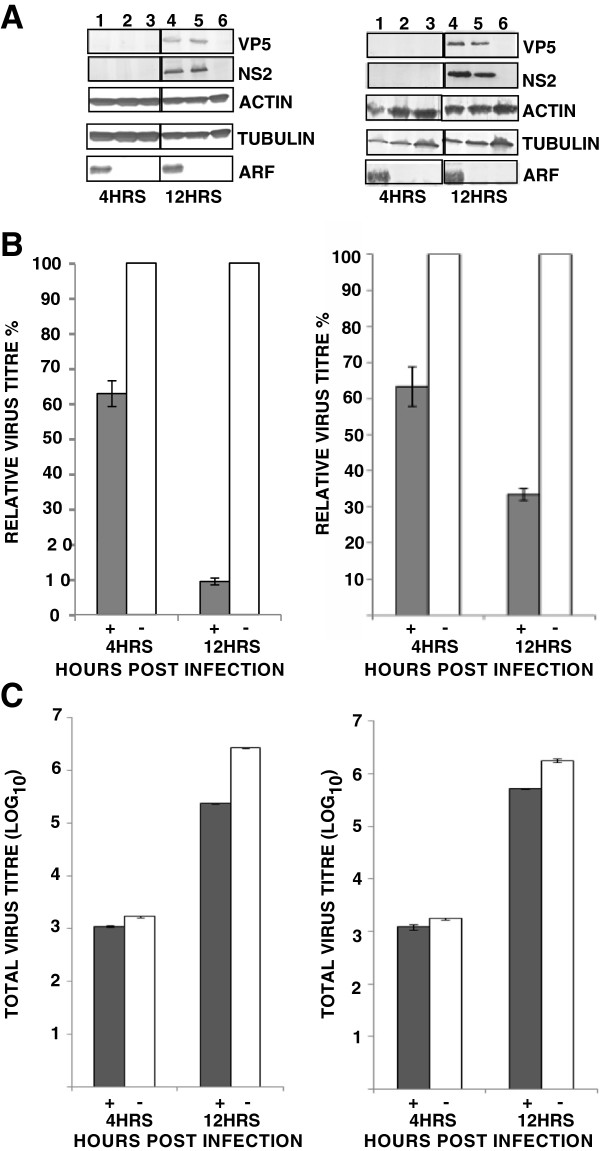
**Effect of Arf6/Q67L induced PI(4,5)P**_**2**_**-enriched vesicles on virus production.** (**A**) Western Blot analysis of HeLa (right) and BSR (left) cells transfected with HA-tagged Arf6/Q67L and infected with BTV-1 were analyzed for expression of viral (VP5 and NS2) and cellular (actin and tubulin) proteins 4 and 12hrs post-infection (lane,1). Controls consist of HeLa cells that have not been transfected but infected with BTV-1 (lane,2) and cells that have neither been transfected nor infected (lane, 3). The proteins were detected by specific antibodies. The western blots were repeated 2 times on three independent experiments. (**B** &**C**) Analysis of relative (**B**) and total (**C**) virus titres in infected HeLa (right) and BSR (left) cells, in the presence (+) or absence (−) of Arf6 mutant Arf6/Q67L. Cells infected with BTV-1 12 hrs post transfection were harvested at 4 and 12 hrs post-infection. For the relative virus tires, the total titre for each time was normalized to 100% for untreated cells. Bars indicate the standard error of three replicates of the experiments.

The effect of Arf6/Q67L on viral protein production and infectious virus titres in HeLa and BSR cells were examined by transfecting with Arf6/Q67L expression plasmid followed by infection with BTV-1. To ensure the expression of the plasmid, confocal microscopy analysis was undertaken which revealed substantial level of Arf6/Q67L expression. When these transfected cells were infected with BTV-1 and viral protein expression were analyzed by WB (repeated 2 times on three independent experiments) at 4 and 12 hrs post-infection, BTV proteins were detected both in HeLa (Figure 6A right) and BSR (Figure 6A left) cells only at 12 hrs but not at 4 hrs post-infection (Figure 6A right & left, lane 1). Control infected cells that were not expressing Arf6/Q67L also showed expression of the viral proteins at the same time of post-infection (Figure 6A right & left, lane 2). Uninfected cells served as negative control (Figure 6A right & left, lane 3). Furthermore, production of cellular proteins (tubulin and actin) was monitored in each case including the transfected and infected cells (Figure 6A right & left lane 1); non transfected but infected cells (Figure 6A right & left, lane 2) and non transfected and uninfected cells (Figure 6A right & left, lane 3). All confirmed that sequestration of PI(4,5)P_2_ did not alter the production of cellular proteins. In all these experiments the expression of HA tagged Arf6/Q67L expression plasmid was also monitored (Figure 6A right & left, lane 1). In addition, infected cells transfected with an unrelated plasmid or treated with transfection reagent showed similar expression profiles of the proteins as the control infected cells without any transfection (data not shown).

The effect of Arf6/Q67L on virus yield was further investigated by infecting the Arf6/Q67L expressing cells and analyzing the total titres by plaque assay as described in Materials and Methods (Figure 6B and C). When the relative titres were compared to control cells (Figure 6B, HeLa, right, and BSR, left) that were infected but not transfected, cells expressing the dominant negative plasmid, Arf6/Q67L showed significant reduction in viral tires at 12 hrs (p < 0.0001 in HeLa and p = 0.0007 in BSR) post-infection, but not at 4 hrs (p = 0.01 in HeLa and p = 0.2 in BSR). A similar trend was also observed for total viral tires (Figure 6C) where the reduction at 12 hrs was more significant (p = 0.003 and 0.001 for HeLa and BSR cells, respectively) than 4 hrs (p > 0.005 for HeLa and BSR) post infection in the both cell types. Since, no viral proteins were expressed at 4 hrs post-infection, this suggested that the viral particles counted at this early time post infection were the input virus particles and not newly assembled ones. Although the percentage of decrease in relative virus titre was more in HeLa cells (90%) than BSR (70%), similar trends in decrease of relative virus titre confirmed that perturbation of cellular PI(4,5)P_2_ inhibits virus production. Thus, the formation of PI(4,5)P_2_ enriched vesicles inhibits virus production but does not interfere with virus protein production.

**Figure 7:**
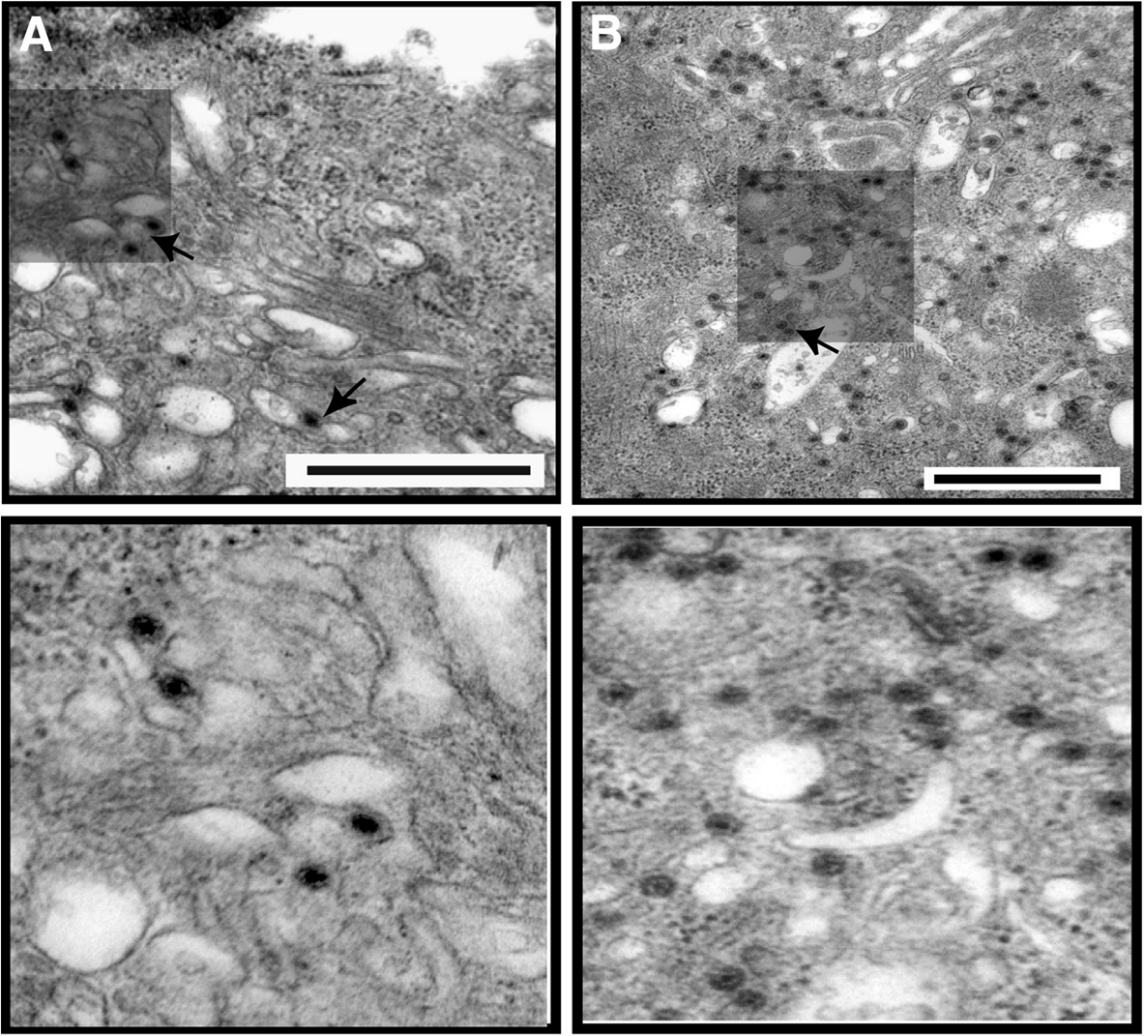
**Analysis of sequestration of PI(4,5)P**_**2 **_**by Arf6/Q67L on BTV particle production by EM.** (**A**). HeLa cells transiently expressing Arf6/Q67L were infected with BTV-1 and analyzed for virus particle production by EM. (**B**). Control consists of cells that have not been transiently transfected with plasmid expressing Arf6/Q67L but infected with BTV-1. The presence of virus particles in cell section (upper panel) is indicated by arrows. Lower panels are magnified section. The bars represent magnification of 500 nm.

Since sequestration of PI(4,5)P_2_ into intra-cytoplasmic vesicles by Arf6/Q67L expression hampered the relative virus production, it was important to determine whether disrupting the distribution of PI(4,5)P_2_ in cells impairs virus particle distribution. When the transfected and infected cells were compared by EM from three different planes of the cells’ sections (Figure 7), the total number of virus particles was significantly less than the non-transfected cells (about 90% less, p = 0.008) (see Figure 7A and 7B). In addition a large number of large vesicles were visible (see Figure 7A). Thus, the EM data further support the hypothesis that PI(4,5)P_2_ expression in cells plays an important role in BTV particle production.

## Discussion

Although BTV is a non-enveloped virus, the outer capsid protein VP5 possesses fusogenic property [5] as well as structural similarity with the fusion proteins of enveloped viruses [7]. In addition, VP5 also possesses a SNARE domain [24] that is very similar to SNARE domains of cellular proteins that have been shown to interact with PI(4,5)P_2_[35,36]. In addition a second BTV protein, NS3 has some functional similarities with HIV Gag [13,14,16] and it also interacts with cellular annexin2 [15], which in turn, interacts with PI(4,5)P_2_ present in membranes [17-22]. These compelling findings pointed at PI(4,5)P_2_ as the common denominator in the interactions between a non-enveloped virus (BTV) and host cells. In the case of enveloped viruses such as HIV, where the virus assembly occurs on the cellular membranes [37-40], the basic domain of HIV matrix protein (MA) has been suggested to contribute to the membrane binding of Gag by interacting with acidic phospholipids on the cytoplasmic leaflet of membranes [31,41,42]. Furthermore, a recent report has also shown that the subcellular localization of Gag in MLV infected cells is also determined by PI(4,5)P_2_[43].

This study therefore focused on the effect of PI(4,5)P_2_ during virus maturation. On the basis of earlier studies that have successively used PH-GFP as a marker for PI(4,5)P_2_ in cells [43], the same lipid marker was also utilized to study the role of the negatively charged lipid in BTV maturation. The experiments undertaken on particle production and protein synthesis were limited up to 12 hrs post-infection as the first replication cycle of BTV infection is completed by 16 hrs post-infection. Additionally, in order to negate whether the effects of lipid is not restricted to one particular cell type, two different cell types were analyzed for the affect of lipid on BTV morphogenesis. The sequestration of VP5 and NS3 to PI(4,5)P_2_-enriched endosomal vesicles by Arf6/Q67L expression and a decrease in relative virus titre in the presence of Arf6/Q67L indicated that disruption in the distribution pattern of PI(4,5)P_2_ hampered virus particle production. In addition since depletion of PI(4,5)P_2_ prior to BTV infection also decreased particle production, the results presented here strongly suggest that PI(4,5)P_2_ plays an important role in the BTV life cycle. As neither depleting the level of PI(4,5)P_2_ nor altering its distribution disrupted the level of viral proteins, this confirmed that although PI(4,5)P_2_ does play an active role in virus assembly, it does not have any role in viral protein production. Since the early time point (i.e., 4 hrs) showed some virus titres but no viral protein production, this indicated that the titres were due to the presence of the input virus. EM sectioning of BTV infected cells exhibited the attachment of viral particles to the outer surface of vesicle-like structures that were absent in PI(4,5)P_2_ depleted cells. Moreover, EM analyses of cells expressing Arf6/Q67L also showed a decrease in virus production. Some studies have reported a co-relation between expression of 5-phosphate IV and apoptosis related decrease in cell viability. However, since our experiments involving confocal microscopy (results not shown) of the BSR and HeLa cells over expressing 5-phosphate IV have not shown any of the gross cellular morphological changes that are usually visible in apoptotic cells, the decrease in virus titer due to reduction of PI(4,5)P_2_ can therefore be attributed to the effect of the absence of the lipid in cells and not due to apoptosis induced by over expression of 5-phosphate IV in transfected cells. In addition, previous research has also confirmed that BTV actively induces apoptosis in infected mammalian cells that does not have any negative impact on virus particle production [44].

It is well established that PI(4,5)P_2_ plays an important role in the generation and trafficking of intra-cytoplasmic vesicles via the cytoskeletal tracks [45,46]. Studies in polarized epithelial cells have revealed that many newly synthesized proteins in the Golgi network, that are destined for the apical surface, are segregated into membrane components rich in sphingolipids and cholesterol [47]. In comparison, proteins destined for the basolateral surface are sorted into vesicles that are predominantly composed of glycerophospholipid. Cells that are not overtly polarized, like the fibroblasts, also deploy these two distinct pathways [48,49]. A study comprising of influenza virus hemagglutinin (HA) established that vesicles containing HA were delivered from the Golgi to the cell surface as the infection progressed in the cells. Based on this, it can be hypothesized that disruption of PI(4,5)P_2_ in cells either by its depletion or altered distribution hampers the generation of the intracytoplasmic vesicles that might act as hubs for BTV assembly in infected cells. This notion can be substantiated by the fact that NS3 interacts with the two outer capsid proteins of BTV, VP2 and VP5 [15,24] and it also plays an essential role in virus egress [13,14].

The combined data obtained from various experiments in this study provide conclusive evidence of the importance of PI(4,5)P_2_ in BTV infection which, to our knowledge, is the first report demonstrating involvement of PI(4,5)P_2_ in a non-enveloped virus assembly and release. It is also possible that the effect of PI(4,5)P_2_ on BTV maturation might also be due to changes in its cellular regulation caused by virus infection or that PI(4,5)P_2_ might affect BTV particle production due to its effect on NS3 and VP5 by perturbing the levels of either annexin-2 or SNARE proteins, the cellular binding partners of the two viral proteins. Further studies will be necessary to clarify this. Based on our current data it can be hypothesized that common elements may underlie the pathways of virus maturation used by both enveloped and non-enveloped viruses alike.

## Conclusion

The principal findings of this research is that PI(4,5)P_2_ influences BTV maturation. In addition this is a unique demonstration of an essential role for negatively charged membrane lipid molecules in the morphogenesis of BTV. It also suggested that the egress pathways of capsid and enveloped viruses may be more closely related than commonly supposed.

## Methods

### Cells and viruses

HeLa (human cervical epithelial) and BSR (a derivative of Baby Hamster Kidney cell) were maintained as described previously [24]. The BSR cells were used to propagate the BTV serotype (BTV-1 SA) and to determine the viral titre by plaque assay. For time course studies of viral infection, HeLa and BSR cell monolayers were washed with FCS-free growth medium and infected with BTV at an MOI of 1. Virus adsorptions were carried out for 30 minutes at 4°C, followed by incubation at 37°C in growth medium supplemented with 2% FCS for 4 and 12 hrs.

### Reagents, buffers and antibodies

Reagents required for protein interaction and confocal microscopy studies were obtained as described previously [24]. Except for VP2 [50], all the antibodies used against BTV proteins were generated in our laboratory. While various antibodies used in this study against the cellular proteins have been described previously [24], the mouse monoclonal anti-myc (9E10), rabbit polyclonal anti-HA and mouse monoclonal anti PI(4,5)P_2_ were obtained from Abcam (Cambridge,UK), Santa Cruz Biotechnology (SantaCruz, USA) and Molecular Probes (USA), respectively.

### Plasmids

Plasmids expressing 5ptaseIV and Δ1 mutant lacking the phosphatase signature domain were donated by P. Majerus (Washington University School of Medicine, St. Louis) and Eric Freed (National Cancer Institute, NIH, Frederick, Maryland) respectively. The PHGFP expression plasmids and HA-tagged Arf6/Q67L were donated by T. Balla (National Institute of Child Health and Human Development, NIH) and J. Donaldson (National Heart, Lung, and Blood Institute, NIH) respectively.

### Transfection

HeLa cells were seeded in 12 well plates, transfected when 70% confluent with Lipofectaminine 2000 (Invitrogen) according to the manufacturer’s recommendations and incubated for 12 hrs at 37°C. Subsequently they were infected with the virus, incubated for the various time points and then processed for titration assays, western blot or confocal microscopy assays as described below.

### Virus titration

Cell extracts from BTV-infected and either transfected or treated with tranfecting reagent were collected, freeze thawed three times and virus titres were determined by plaque assays using BSR cells as described previously [51]. The total viral titer was determined and normalized to the titer obtained for infected but untransfected cells. The mean and standard error of the reduction mediated by the inhibitor were calculated (Sigma Plot 2000; Systat Software Inc.).

### Western blot

SDS-PAGE separated proteins were transferred onto a Hybond enhanced chemiluminescence nitrocellulose membrane (GE Healthcare, Uppsala Sweden) and probed with appropriate antibodies. Subsequently, the blots were incubated with alkaline phosphatase conjugated secondary antibodies and developed with BCIP-NBT substrate (Sigma-Aldrich). The western blots were repeated 2 times on three independent experiments.

### Confocal microscopy

Mammalian cells were seeded in 24 well plates on 13-mm-diameter coverslips, transfected with the expression plasmids and infected with BTV. Subsequently the cells were processed for confocal microscopy as described previously [52]. After analyzing with a Zeiss LSM 510 confocal microscope, the images were obtained using LSM 510 image browser software and processed using Photoshop Elements 2.0 software (Adobe).

### Electron microscopy (EM)

HeLa cells were transfected with the expression plasmids followed by infection with virus and incubated for 12 hrs at 37°C. The cells were then processed for EM as described previously [14] and examined by a Hitachi H7000 electron microscope. The experiment was repeated twice and 3 different sections per independent experiment were analyzed for the distribution of virus particles. The statistical analysis of the virus particles were undertaken by Sigma Plot 2000 (Systat Software Inc.) and Excel (Microsoft).

## Competing interests

The authors declare that they have no competing interests.

## Authors’ contributions

BB conceived, designed and performed the experiments. BB and PR analyzed the data. BB and PR wrote the manuscript. All authors read and approved the final manuscript.

## References

[B1] Roy P Noad R Bluetongue virus assembly and morphogenesis Curr Top Microbiol Immunol 2006 309 87 116 10.1007/3-540-30773-7_4 16909898

[B2] Eaton BT Crameri GS The site of bluetongue virus attachment to glycophorins from a number of animal erythrocytes J Gen Virol 1989 70 3347 3353 10.1099/0022-1317-70-12-3347 2558161

[B3] Hassan SH Roy P Expression and functional characterization of bluetongue virus VP2 protein: Role in cell entry J Virol 1999 73 9832 9842 10559295 10.1128/jvi.73.12.9832-9842.1999 PMC113032

[B4] Hassan SH Wirblich C Forzan M Roy P Expression and functional characterization of bluetongue virus VP5 protein: role in cellular permeabilization J Virol 2001 75 8356 8367 10.1128/JVI.75.18.8356-8367.2001 11507181 PMC115081

[B5] Forzan M Wirblich C Roy P A capsid protein of nonenveloped Bluetongue virus exhibits membrane fusion activity PNAS 2004 101 2100 2105 10.1073/pnas.0306448101 14762165 PMC357058

[B6] Forzan M Marsh M Roy P Bluetongue virus entry into cells J Virol 2007 81 4819 4827 10.1128/JVI.02284-06 17267479 PMC1900141

[B7] Zhang X Boyce M Bhattacharya B Zhang X Scheina S Roy P Zhou ZH Bluetongue virus coat protein VP2 contains a sialic acid-binding domain and VP5 has similarities to enveloped virus fusion proteins PNAS 2010 107 6292 6297 10.1073/pnas.0913403107 20332209 PMC2852009

[B8] Modrof J Lymperopoulos K Roy P Phosphorylation of Bluetongue Virus Nonstructural Protein 2 Is Essential for Formation of Viral Inclusion Bodies J Virol 2005 79 10023 10031 10.1128/JVI.79.15.10023-10031.2005 16014962 PMC1181561

[B9] Kar AK Ghosh M Roy P Mapping the assembly of Bluetongue virus scaffolding protein VP3 Virology 2004 324 387 399 10.1016/j.virol.2004.04.018 15207624

[B10] Kar AK Bhattacharya B Roy P Bluetongue virus RNA binding protein NS2 is a modulator of viral replication and assembly BMC Mol Biol 2007 8 4 10.1186/1471-2199-8-4 17241458 PMC1794256

[B11] Wu X Chen SY Iwata H Compans RW Roy P Multiple glycoproteins synthesized by the smallest RNA segment (S10) of bluetongue virus J Virol 1992 66 7104 7112 1331513 10.1128/jvi.66.12.7104-7112.1992 PMC240390

[B12] Hyatt AD Zhao Y Roy P Release of bluetongue virus-like particles from insect cells is mediated by BTV nonstructural protein NS3/NS3A Virology 1993 193 592 603 10.1006/viro.1993.1167 8384747

[B13] Wirblich C Bhattacharya B Roy P Nonstructural protein 3 of bluetongue virus assists virus release by recruiting ESCRT-I protein Tsg101 J Virol 2006 80 460 473 10.1128/JVI.80.1.460-473.2006 16352570 PMC1317520

[B14] Celma CC Roy P A viral nonstructural protein regulates bluetongue virus trafficking and release J Virol 2009 83 6806 6816 10.1128/JVI.00263-09 19369335 PMC2698550

[B15] Beaton AR Rodriguez J Reddy YK Roy P The membrane trafficking protein calpactin forms a complex with bluetongue virus protein NS3 and mediates virus release Proc Natl Acad Sci USA 2002 99 13154 13159 10.1073/pnas.192432299 12235365 PMC130602

[B16] Celma CC Roy P Interaction of calpactin light chain (S100A10/p11) and a viral NS protein is essential for intracellular trafficking of nonenveloped bluetongue virus J Virol 2011 85 4783 4791 10.1128/JVI.02352-10 21411520 PMC3126219

[B17] Ayala-Sanmartin J Henry JP Pradel LA Cholesterol regulates membrane binding and aggregation by annexin 2 at submicromolar Ca(2+) concentration Biochim Biophys Acta 2001 1510 18 28 10.1016/S0005-2736(00)00262-5 11342144

[B18] Hayes MJ Merrifield CJ Shao D Ayala-Sanmartin J Schorey CD Levine TP Proust J Curran J Bailly M Moss SE Annexin A2 binding to phosphatidylinositol 4,5-bisphosphate on endocytic vesicles is regulated by the stress response pathway J Biol Chem 2004 279 14157 14164 10.1074/jbc.M313025200 14734570 PMC1351152

[B19] Rescher U Ruhe D Ludwig C Zobiack N Gerke V Annexin 2 is a phosphatidylinositol (4,5)-bisphosphate binding protein recruited to actin assembly sites at cellular membranes J Cell Sci 2004 117 3473 3480 10.1242/jcs.01208 15226372

[B20] Chasserot-Golaz S Vitale N Umbrecht-Jenck E Knight D Gerke V Bader MF Annexin 2 promotes the formation of lipid microdomains required for calcium-regulated exocytosis of dense-core vesicles Mol Biol Cell 2005 16 1108 1119 10.1091/mbc.E04-07-0627 15635098 PMC551477

[B21] Gokhale NA Abraham A Digman MA Gratton E Cho W Phosphoinositide specificity of and mechanism of lipid domain formation by annexin A2-p11 heterotetramer J Biol Chem 2005 280 42831 42840 10.1074/jbc.M508129200 16230353

[B22] Volker G Creutze CE Moss SE Annexins: linking Ca2+ signalling to membrane dynamics Nat Rev Mol Cell Biol 2005 6 449 461 10.1038/nrm1661 15928709

[B23] Somanath S Barg S Marshall C Silwood CJ Turner MD High extracellular glucose inhibits exocytosis through disruption of syntaxin 1A-containing lipid rafts Biochem Biophys Res Commun 2009 389 241 246 10.1016/j.bbrc.2009.08.126 19716806

[B24] Bhattacharya B Roy P Bluetongue virus outer capsid protein VP5 interacts with membrane lipid rafts via a SNARE domain J Virol 2008 27 27 10.1128/JVI.01274-08 PMC2573168 18753209

[B25] Simonsen A Wurmser A Emr SD Stenmark H The role of phosphoinositides in membrane transport Curr Opin Cell Biol 2001 13 485 492 10.1016/S0955-0674(00)00240-4 11454456

[B26] Donaldson JG Multiple roles for Arf6: sorting, structuring, and signaling at the plasma membrane J Biol Chem 2003 278 41573 41576 10.1074/jbc.R300026200 12912991

[B27] Brown FD Rozelle AL Yin HL Balla T Donaldson JG Phosphatidylinositol 4,5-bisphosphate and Arf6-regulated membrane traffic J Cell Biol 2001 154 1007 1017 10.1083/jcb.200103107 11535619 PMC2196179

[B28] Aikawa Y Martin TF ARF6 regulates a plasma membrane pool of phosphatidylinositol(4,5)bisphosphate required for regulated exocytosis J Cell Biol 2003 162 647 659 10.1083/jcb.200212142 12925709 PMC2173784

[B29] Várnai P Bala T Visualization of phosphoinositides that bind pleckstrin homology domains: calcium- and agonist-induced dynamic changes and relationship to myo-[3H]inositol-labeled phosphoinositide pools J Cell Biol 1998 143 501 510 10.1083/jcb.143.2.501 9786958 PMC2132833

[B30] Kisseleva MV Wilson MP Majerus PW The isolation and characterization of a cDNA encoding phospholipid-specific inositol polyphosphate 5-phosphatase J Biol Chem 2000 275 20110 20116 10.1074/jbc.M910119199 10764818

[B31] Ono A Ablan SD Lockett SJ Nagashima K Freed EO Phosphatidylinositol (4,5) bisphosphate regulates HIV-1 Gag targeting to the plasma membrane PNAS 2004 101 14889 14894 10.1073/pnas.0405596101 15465916 PMC522033

[B32] Kar AK Iwatani N Roy P Assembly and intracellular localization of the bluetongue virus core protein VP3 J Virol 2005 79 11487 11495 10.1128/JVI.79.17.11487-11495.2005 16103199 PMC1193605

[B33] Kisseleva MV Cao L Majerus PW Phosphoinositide-specific inositol polyphosphate 5-phosphatase IV inhibits Akt/Protein Kinase B phosphorylation and leads to apoptotic cell death J Biol Chem 2002 277 6266 6272 10.1074/jbc.M105969200 11706019

[B34] Honda A Nogami M Yokozeki T Yamazaki M Nakamura H Watanabe H Kawamoto K Nakayama K Morris AJ Frohman MA Kanaho Y Phosphatidylinositol 4-phosphate 5-kinase alpha is a downstream effector of the small G protein ARF6 in membrane ruffle formation Cell Biol 1999 99 521 532 10.1016/s0092-8674(00)81540-8 10589680

[B35] Holz RW Hlubek M Sorensen SD Fisher SK Balla T Ozaki S Prestwich GD Stuenkel EL Bittner MA A pleckstrin homology domain specific for phosphatidylinositol 4,5-bisphosphate (PtdIns-4,5-P2) and fused to green fluorescent protein identifies plasma membrane PtdIns-4,5-P2 as being important in exocytosis J Biol Chem 2000 275 17878 17885 10.1074/jbc.M000925200 10747966

[B36] Bai J Tucker WC Chapman ER PIP2 increases the speed of response of synaptotagmin and steers its membrane-penetration activity toward the plasma membrane Nat Struct Mol Biol 2004 11 36 44 10.1038/nsmb709 14718921

[B37] Raposo G Moore M Innes D Leijendekker R Leigh-Brown A Benaroch P Geuze H Human macrophages accumulate HIV-1 particles in MHC II compartments Traffic 2002 3 718 729 10.1034/j.1600-0854.2002.31004.x 12230470

[B38] Pelchen-Matthews A Kramer B Marsh M Infectious HIV-1 assembles in late endosomes in primary macrophages J Cell Biol 2003 162 443 455 10.1083/jcb.200304008 12885763 PMC2172706

[B39] Nydegger S Foti M Derdowski A Spearman P Thali M HIV-1 egress is gated through late endosomal membranes Traffic 2003 4 902 910 10.1046/j.1600-0854.2003.00145.x 14617353

[B40] Sherer NM Lehmann MJ Jimenez-Soto LF Ingmundson A Horner SM Cicchetti G Allen PG Pypaert M Cunningham JM Mothes W Visualization of retroviral replication in living cells reveals budding into multivesicular bodies Traffic 2003 4 785 801 10.1034/j.1600-0854.2003.00135.x 14617360

[B41] Zhou W Parent LJ Wills JW Resh MD Identification of a membrane-binding domain within the amino-terminal region of human immunodeficiency virus type 1 Gag protein which interacts with acidic phospholipids J Virol 1994 68 2556 2569 8139035 10.1128/jvi.68.4.2556-2569.1994 PMC236733

[B42] Chukkapalli V Hogue IB Boyko V Hu WS Ono A Interaction between the human immunodeficiency virus type 1 gag matrix domain and phosphatidylinositol-(4,5)-bisphosphate is essential for efficient gag membrane binding J Virol 2008 82 2405 2417 10.1128/JVI.01614-07 18094158 PMC2258911

[B43] Hamard-Peron E Juillard F Saad JS Roy C Roingeard P Summers MF Darlix JL Picart C Muriaux D Targeting of murine leukemia virus gag to the plasma membrane is mediated by PI(4,5)P2/PS and a polybasic region in the matrix J Virol 2010 84 503 515 10.1128/JVI.01134-09 19828619 PMC2798412

[B44] Stewart ME Roy P Role of cellular caspases, nuclear factor-kappa B and interferon regulatory factors in Bluetongue virus infection and cell fate Virol J 2010 7 362 10.1186/1743-422X-7-362 21134281 PMC3002312

[B45] Kanzaki M Furukawa M Raab W Pessin JE Phosphatidylinositol 4,5-bisphosphate regulates adipocyte actin dynamics and GLUT4 vesicle recycling J Biol Chem 2004 279 30622 30633 10.1074/jbc.M401443200 15123724

[B46] Cremona O Di Paolo G Wenk MR Lüthi A Kim WT Takei K Daniell L Nemoto Y Shears SB Flavell RA McCormick DA De Camilli P Essential role of phosphoinositide metabolism in synaptic vesicle recycling Cell 1999 99 179 188 10.1016/S0092-8674(00)81649-9 10535736

[B47] Harder TS Simons K Caveolae, DIGs and the dynamics of sphingolipid-cholesterol microdomains Curr Opin Cell Biol 1997 9 534 542 10.1016/S0955-0674(97)80030-0 9261060

[B48] Yoshimori T Keller P Roth MG Simons K Different biosynthetic transport routes to the plasma membrane in BHK and CHO cells J Cell Biol 1996 133 247 256 10.1083/jcb.133.2.247 8609159 PMC2120802

[B49] Harder T Scheiffele P Verkade P Simons K Lipid domain structure of the plasma membrane revealed by patching of membrane components J Cell Biol 1998 141 929 942 10.1083/jcb.141.4.929 9585412 PMC2132776

[B50] DeMaula CD Heidner HW Rossitto PV Pierce CM MacLachlan NJ Neutralization determinants of United States bluetongue virus serotype 10 Virology 1993 195 292 296 10.1006/viro.1993.1377 7686312

[B51] Bhattacharya B Roy P Role of lipids on entry and exit of bluetongue virus, a complex non-enveloped virus Viruses 2010 2 1218 1235 10.3390/v2051218 21994677 PMC3187602

[B52] Bhattacharya B Noad RJ Roy P Interaction between Bluetongue virus outer capsid protein VP2 and vimentin is necessary for virus egress Virol J 2007 4 7 10.1186/1743-422X-4-7 17224050 PMC1783847

